# How and for Whom Is Mobile Phone Addiction Associated with Mind Wandering: The Mediating Role of Fatigue and Moderating Role of Rumination

**DOI:** 10.3390/ijerph192315886

**Published:** 2022-11-29

**Authors:** Shuailei Lian, Xuqing Bai, Xiaowei Zhu, Xiaojun Sun, Zongkui Zhou

**Affiliations:** 1 College of Education and Sports Science, Yangtze University, Jingzhou 434023, China; 2 Key Laboratory of Adolescent Cyberpsychology and Behavior (CCNU), Ministry of Education, Wuhan 430079, China; 3 School of Psychology, Central China Normal University, Wuhan 430079, China

**Keywords:** mobile phone addiction, mind wandering, fatigue, rumination, executive control failure hypothesis, resource-control theory of mind-wandering

## Abstract

With the increasing prevalence of mobile phone addiction, mobile phone addiction has been considered a prominent risk factor for internalizing or externalizing problems, such as psychological distress and irrational procrastination. However, few studies shed light on the effect of mobile phone addiction on mind wandering and the underlying mechanisms. This study speculated that the direct effect of mobile phone addiction on mind wandering may be linked to fatigue and that the level of an individual’s personality characteristics, such as rumination, may influence both the direct and indirect effects of mobile phone addiction on mind wandering. To test these hypotheses, we recruited 1811 college students to complete the self-report questionnaires. The results indicated that mobile phone addiction was positively associated with mind wandering. This direct effect could be mediated by fatigue, and both the direct and indirect effects of mobile phone addiction on mind wandering could be moderated by rumination. Specifically, both the direct and indirect effects were stronger for students with high rumination. These findings enrich our understanding of how, why, and for whom mobile phone addiction is correlated with mind wandering.

## 1. Introduction

With the development of information technology, the functions of mobile phones are increasingly becoming diversified. The mobile phone, as the most visible product of the information technology revolution, has profoundly changed people’s work and lifestyles, as well as shaped a new political and economic life form. Mobile phones not only make mobile offices, learning, interpersonal communication, financial management, and payment more convenient, but also make people’s leisure and entertainment more diverse and colorful. Due to the diversification of mobile phone features and the perceived ease of use of mobile phones, mobile phones have won the favor of all age groups and have a high penetration rate in all age groups [1], especially in college students [2]. According to the survey by the Pew Research Center, 90% of the global population owns mobile phones, including 68% of the Chinese [3]. As this rate increases year by year, 99.7% of the 1.032 billion Internet users (1.029 billion) will have mobile phones as of December 2021 [4]. However, driven by “crisis consciousness” or “negative automatic thinking” the dark side of the relationship between people and mobile phones, mobile phone addiction, or problematic mobile phone use, has attracted great attention [5,6]. Mobile phone addiction refers to an addictive behavior in which individuals excessively and compulsively use mobile phones, resulting in negative effects on their psychological, behavioral, and social functions [7]. Previous studies demonstrated that although mobile phones have brought many conveniences to individuals’ lives, the convenience and high user stickiness of mobile phones also increase the possibility of individuals becoming addicted to mobile phones [5,8,9]. Therefore, the adverse impact of mobile phone addiction on individuals’ mental health has become the focus of researchers’ attention.

Previous studies have examined the negative effects of mobile phone addiction on mental health and have yielded a wealth of research findings. First, as a kind of behavioral addiction, mobile phone addiction has a serious negative impact on individuals’ behavior adaptation. For example, Lian et al., [10] demonstrated that mobile phone addiction was an important reason for individuals’ irrational procrastination. Second, as a kind of behavior adaptation problem, mobile phone addiction could also make individuals fall into serious emotional adaptation problems. For instance, Lian et al. [11] showed that mobile phone addiction could induce individuals’ ruminative responses and cause psychological distress. This conclusion has also been confirmed by many studies [12,13,14]. Besides, studies also revealed a negative relationship between mobile phone addiction and sleep quality [15,16], as well as a positive relationship between mobile phone addiction and cognitive failures [17,18]. As a mobile communication device with strong user stickiness, mobile phones were bound to have an impact on the individual’s state of attention or consciousness, especially for individuals with mobile phone addiction [19]. A few studies have found that individuals who are addicted to the Internet and Internet gaming have inattention problems, such as mind wandering [20,21]. However, few studies focus on the association between mobile phone addiction and individuals’ attention quality and state of consciousness. The mediating mechanisms and contextual factors underlying this link were also unclear. To fill this gap, based on the executive-control failure hypothesis [22,23] and resource-control theory of mind wandering [24], the present study will detect the relationship between mobile phone addiction and mind wandering as well as take fatigue as a mediator and rumination as a moderator to reveal the mediation and moderation mechanisms that link mobile phone addiction with mind wandering. The results of this study will not only help us understand the psychological mechanism of mobile phone addiction leading to individuals’ mind wandering comprehensively and profoundly, but also reveal the potential individual differences in this psychological mechanism. The findings of this study will also sound an alarm for mobile phone users to avoid excessive use of mobile phones and the potential adverse effects of non-adaptive mobile phone use (mobile phone addiction) on their attention quality or state of consciousness.

### 1.1. Mobile Phone Addiction and Mind Wandering

Mind wandering, as a common conscious experience, refers to a state of consciousness generated spontaneously when one is awake, during which the endogenous psychological representation is realized by individuals uncontrollably without explicit goal guidance [25]. In a recent survey, the incidence of mind wandering was about 40% [26]. Mind wandering has been considered an ineffective or even negative psychological process, as it could make individuals unable to concentrate on the current task and decrease their behavioral performance [25,27]. Therefore, mind wandering has been widely concerned by researchers and has been given different names in different problem situations, such as daydreaming [28], task-unrelated thought (TUT) [29], stimulus independent thought (SIT) [30], spontaneous thought process [31], and off-task thinking [32]. The content and inducing factors of mind wandering have also attracted extensive attention in previous studies, such as negative emotions [33], fatigue [34], and rumination [35,36]. As mobile information technology develops rapidly, the mobile phone, as one of the most attractive mobile internet terminals, has become a major killer of individuals’ attention quality [19,37,38]. It is worth emphasizing that mobile phones are a collection of functions such as the Internet and Internet games, which may make individuals addicted to mobile phones and also have similar symptoms and negative effects (e.g., mind wandering) of related addiction problems to a certain extent [20,21]. Mobile phone addiction may also be a prominent risk factor leading to mind wandering. Previous studies demonstrated that mobile phone addiction was positively related to a variety of emotional adaptation problems, such as depression, anxiety, and stress [11,39]. Negative emotions including depression, anxiety, and stress was an important inducement to induce individuals’ mind wandering [27,33,40]. Therefore, individuals suffering from mobile phone addiction may be more likely to engage in mind wandering due to the negative emotions drawn from excessive or compulsive mobile phone use. Prior studies have also shown that mobile phone addiction could not only lead to insufficient sleep time, but also may result in poor sleep quality and even sleep disorders [27,33,40]. Adequate sleep time and high sleep quality are important guarantees for individuals to maintain good attention quality and avoid mind wandering. Therefore, individuals suffering from mobile phone addiction may have poor attention quality and a high frequency of mind wandering due to their poor sleep quality. Besides, Lian and his colleagues [37] showed that mobile phone addiction was negatively associated with college students’ attention control. Considering that mind wandering is the consequence of attention or executive control failure [22], mobile phone addiction may have a positive association with mind wandering. Moreover, an experimental study showed that mobile phone addicts could hardly maintain their goals in a continuous performance task (AX-CPT), which indicated that mobile phone addiction was positively associated with executive-control failure [37]. According to the executive-control failure hypothesis, the main cause of mind wandering is a failure of executive control [22,23]. Therefore, mobile phone addiction may contribute to mind wandering.

Although many indirect studies imply a positive correlation between mobile phone addiction and mind wandering, it is unclear how and for whom mobile phone addiction leads to mind wandering. Therefore, this study took fatigue as a mediator and rumination as a moderator and conducted a moderated mediation model to reveal the mechanisms linking mobile phone addiction and mind wandering.

### 1.2. Fatigue as a Mediator

According to the executive-control failure hypothesis and resource-control theory of mind-wandering, executive control failure is the core cause of mind wandering [22,23,24]. Fatigue may be an important factor affecting executive-control resources in mobile phone addicts. Fatigue, as a complex concept, has been defined in different ways in different disciplines such as psychology, management, and health sciences [41,42,43]. Most researchers consider fatigue to be a subjective feeling of exhaustion caused by the combined interaction of physiological and psychological factors [42,43]. Previous studies demonstrated that workload, negative affect, and poor sleep quality were all positively associated with fatigue [44,45,46]. Whereas, psychological detachment, positive affect showed a negative correlation with fatigue [45]. With the popularity of mobile communication devices, the relationship between individuals’ behaviors or activities in cyberspace and fatigue has also attracted the attention of researchers. Prior studies illustrated that too much time spent on social media or Facebook, social network site addiction, or social comparison on Facebook could make individuals feel tired, exhausted, or other states of fatigue [10,47,48,49,50].

Previous studies have also implied a positive relationship between mobile phone addiction and fatigue. First, a previous study demonstrated that mobile phone addiction was an important reason for the decline of sleep quality in the era of mobile Internet [15]. Given that poor sleep quality is positively related to fatigue, mobile phone addiction may also show a positive correlation with fatigue. Second, prior studies have found that mobile phone addiction could increase the possibility of individuals experiencing depression, anxiety, stress, and other negative affects [11,39]. Negative affect has been proved to be positively related to fatigue [46]. Therefore, negative affect derived from mobile phone abuse may bridge the link between mobile phone addiction and fatigue. Besides, excessive or compulsive use of mobile phones could lead to physical fatigue in the cervical spine, shoulders, finger joints, arms, and other body parts [51,52]. Moreover, the large amount of information communication carried out by mobile phone addicts will increase their cognitive load and make them feel fatigued [43]. There was also a study based on Chinese college students that revealed a positive relationship between mobile phone addiction and fatigue [53]. Therefore, mobile phone addiction may be positively associated with fatigue.

Fatigue may also be a key cause of mind wandering. Previous studies have concluded that fatigue could induce mind wandering [29,33,40,54]. Specifically, these studies found that as the time spent on the task became longer, it became increasingly difficult for participants to accurately maintain their attention on the task. Both the error rate on the task and task-unrelated thoughts would increase with the duration of the task. This fatigue effect in behavior experiments implied that fatigue showed a positive association with mind wandering. Besides, fatigue could lead to the decline of individuals’ executive control resources [55]. According to the resource-control theory of mind-wandering [24], the individual’s self-generated thoughts are the individual’s default state and need to be suppressed by executive control resources. Executive control is an important psychological resource for individuals to maintain a good state of attention and avoid distractions. Conversely, in people with insufficient executive control resources, the mind may wander rather than focus on their target task [22,23]. Thus, fatigue may lead to mind wandering by reducing an individual’s executive control resources. Moreover, a prior study also demonstrated that fatigue may be a mediating factor in the link between mobile phone addiction and psychological adaptation (depressive symptoms) [53].

### 1.3. Rumination as a Moderator

Rumination, as a relatively stable response style, refers to a series of response styles, including chewing and experiencing the negative experience repeatedly, thinking about the causes and potential adverse consequences of the negative experience compulsively, but paying little attention to the potential feasible strategies and measures to improve or solve the negative life events [56]. It is widely regarded as a negative psychological trait that moderates the relationship between risk factors and individuals’ psychological adaptation [57,58,59]. Given that mobile phone addiction has become a prominent risk factor in the era of mobile Internet, rumination may be a moderator factor in the relationship between mobile phone addiction and internalizing and externalizing problems such as depression, anxiety, stress, cognitive failure, and irrational procrastination [11,13,17,18]. In other words, the intensity of both these two links may vary with individuals’ different levels of rumination.

Rumination may accelerate the process of mobile phone addiction, resulting in fatigue. Individuals with high levels of rumination may have poorer sleep quality or sleep disorders when suffering from mobile phone addiction than those with low levels of rumination and thus experience more fatigue. Prior studies have revealed the adverse effect of mobile phone addiction or rumination on individuals’ sleep quality [15,18,60]. According to the main viewpoint of the cumulative model of risk factors [61], the superposition of multiple risk factors (e.g., mobile phone addiction and rumination) will have more serious adverse effects on individuals’ psychological adaptation (e.g., sleep quality). Mobile phone addicts with high levels of rumination not only seriously waste sleep time due to excessive mobile phone use at bedtime but also may be involved in poorer sleep quality or sleep disorders because of their repeatedly ruminating about mobile phone-related activities and experiences before sleeping. Given that poor sleep or sleep disorders were the main cause of fatigue [44], mobile phone addicts with high levels of rumination may have more fatigue due to their poor sleep or sleep disorders after excessive or compulsive use of mobile phones and ruminating more about mobile phone-related activities and experiences in bed. Besides, rumination may strengthen the link between mobile phone addiction and fatigue by catalyzing or amplifying the effects of mobile phone addiction on internalizing problems, such as depression, anxiety, and stress, which are positively associated with fatigue. Previous studies have demonstrated that individuals suffering from mobile phone addiction have more feelings of depression, anxiety, and stress [37,39], and rumination acts as a moderator in this process [53]. Considering that internalizing problems have been proven to be positively correlated with fatigue, the link between mobile phone addiction and fatigue may also be strengthened by rumination.

Rumination may also exacerbate the negative effect of mobile phone addiction on individuals’ attention quality, with mind wandering being more frequent for mobile phone addicts with high levels of rumination. Mobile phone addicts with high levels of rumination may ruminate more about mobile phone-associated activities or experiences. According to the executive-control failure hypothesis [22,23], mind wandering, which represents a failure in executive control, is actually the spontaneous thinking of individuals in response to external experience (mobile phone-associated experience) and inner clues (inner feelings drawn from excessive use of mobile phones). Therefore, mobile phone addicts with high levels of rumination may fall into more mind wandering about mobile phone-related external experiences or inner feelings. Besides, as mentioned earlier, rumination can act as a moderator to exacerbate internalized problems caused by mobile phone addiction, such as depression [53]. Internalizing problems or negative emotions have automatic positive associations with mind wandering [62]. Therefore, mobile phone addicts with high levels of rumination may also be involved in more internalizing problems and thus have more mind wandering. Moreover, according to response style theory [56], individuals with high rumination levels are less likely to notice current things but to let their thoughts wander into their own experiences or feelings repeatedly and involuntarily.

### 1.4. The Present Study

**Figure 1:**
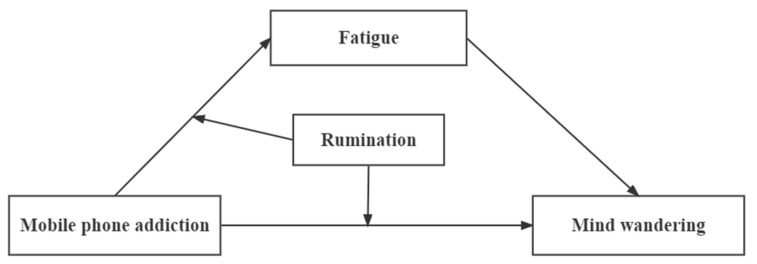
Hypothetical model for this study.

There have been few studies examining the potential adverse effects of mobile phone addiction on individuals’ attention quality. As an important indicator of an individual’s sustained attention, mind wandering is the product of the spontaneity of individual thinking [24]. Similar to individuals with compulsive Internet use and Internet gaming addiction, individuals with mobile phone addiction may also have mind wandering [20,21]. At the same time, according to the executive control failure hypothesis and the resource-control theory of mind-wandering [24], the lack of executive control resources (such as fatigue) is an important reason for the individual’s mind wandering. Negative problems caused by mobile phone addiction, such as lack of sleep and negative emotions, will make individuals experience fatigue [11,15,39], which may be associated with mind wandering [29,33,40,54]. In addition, different individuals may have different resources to cope with fatigue and inhibit mind wandering [24], and rumination may be the key boundary factor [11,13,17,18]. Therefore, this study took mind wandering as an indicator of attention quality and examined the relationship between mobile phone addiction and mind wandering. On this basis, the current study attempted to answer the questions about how and for whom mobile phone addiction can affect individuals’ mind wandering by examining the mediating effect of fatigue and the moderating effect of rumination in the relationship between mobile phone addiction and mind wandering (Figure 1). The research hypotheses were as follows:

 **Hypothesis 1.**  *Mobile phone addiction will be positively associated with mind wandering*.

 **Hypothesis 2.**  *Fatigue will mediate the process of mobile phone addiction linked to mind wandering*.

 **Hypothesis 3a.**  *Rumination will moderate the link between mobile phone addiction and fatigue as well as the indirect effect of fatigue in the association between mobile phone addiction and mind wandering; these effects will be stronger for individuals with higher rumination*.

 **Hypothesis 3b.**  *Rumination will moderate the link between mobile phone addiction and mind wandering, this effect will be stronger for individuals with higher rumination*.

## 2. Materials and Methods

### 2.1. Participants

Through convenient sampling and survey posters, 1811 college students (63.34% female) were recruited at three universities in three cities to complete questionnaires measuring their mobile phone addiction, mind wandering, fatigue, and ruminative response. Two of these universities (Xihua and Jingzhou) are located in southwestern China, and one (Hangzhou) is located in southeastern China. The average age of the participants was 19.74 (*SD* = 1.295). The participants ranged in years of mobile phone usage from 1 to 12 years, with an average age of 5.28 (*SD* = 2.356). Participants included 516 1st-year students (28.49%), 544 s year students (30.04%), and 751 third year students (41.47%).

### 2.2. Procedure

The research design was approved by the institutional ethical committee of the corresponding author’s university. Before the questionnaire was distributed, investigators who had received unified and professional training emphasized the principles of this study, such as anonymity, independence, and confidentiality. Participants gave written consent for participation and then completed the self-reported questionnaire.

### 2.3. Measurements

#### 2.3.1. Mobile Phone Addiction

The Mobile Phone Addiction Scale developed by Leung [63] was employed to assess the degree to which all participants were addicted to mobile phones (e.g., “You never feel like you spend enough time on your phone”). This scale was divided into four dimensions: withdrawal or escape, anxiety or craving, losing control or receiving complaints, and productivity loss. It consisted of 17 items, rated on a 5-point Likert scale from 1 (never) to 5 (always). Higher scores indicate higher degree of mobile phone addiction. This scale has shown good reliability and validity in Chinese college students [64,65]. Cronbach’s α for this scale was 0.846.

#### 2.3.2. Mind Wandering

A Chinese version of the mind wandering scale, revised from the mind wandering scale used by Carriere et al. [66], was adopted to assess the degree of mind wandering among participants (e.g., “I find that I tend to wander unconsciously”). This scale consists of eight items, which require participants to respond on a seven-point Likert-type scale (1 = never, 7 = always). With higher scores reflecting a higher frequency of mind wandering in their daily lives. Confirmatory factor analysis results show that this scale has a good fit: *χ*^2^/*df* = 3.544, RMSEA = 0.037, CFI = 0.992, NFI = 0.989, GFI = 0.993. Cronbach’s α for this scale was 0.848.

#### 2.3.3. Fatigue

The Fatigue Assessment Scale (FAS), developed by Michielsen et al. [67], was used to measure the fatigue level of the participants (e.g., “When I am doing something, I can concentrate quite well”). This scale consisted of 10 items, rated on a 5-point Likert scale from 1 (never) to 5 (always). Higher scores mean high levels of fatigue in everyday life. This scale has shown good reliability and validity among Chinese college students [53]. Cronbach’s α for this scale in the present study was 0.857.

#### 2.3.4. Rumination

The Chinese short version of the Ruminative Response Scale [59] was adopted to measure the ruminative response of participants (e.g., “Think about how sad you feel”). This scale consisted of 10 items, rated on a 4-point Likert scale from 1 (never) to 4 (always). The average score on all items indicates the degree of participants’ ruminative response to the negative life experience. This scale was revised by Treynor and colleagues [56] and showed good reliability and validity in Chinese college students [53]. Cronbach’s α for this scale was 0.821.

#### 2.3.5. Control Variables

In order to control for other variables that have been found to be relevant [11,68,69,70,71], this study used age, gender, and years of mobile phone use as control variables.

### 2.4. Statistical Analyses

First, all observed variables were tested using descriptive statistics and Pearson correlation analysis. Second, the SPSS macro PROCESS (model 4) was adopted to examine the mediating role of fatigue. Moreover, the SPSS macro PROCESS (model 8) was employed to investigate the moderating role of rumination. In order to demonstrate more clearly the moderating effect of rumination, the simple slope analyses suggested by Aiken and West [72] were adopted to dissect the significant interaction effects of rumination.

## 3. Results

### 3.1. Preliminary Analyses

The results of descriptive statistics and Pearson correlations are presented in Table 1. Age was positively associated with years of mobile phone usage and negatively associated with mobile phone addiction. Age, on the other hand, had no significant association with fatigue, mind wandering, or rumination. Years of mobile phone usage were positively associated with mobile phone addiction. However, years of mobile phone usage had no significant association with the other variables. Mobile phone addiction, fatigue, mind wandering, and rumination were positively correlated with each other.

### 3.2. Testing for the Mediating Effect of Fatigue

The SPSS macro PROCESS (model 4) was conducted to examine the mediating effect of fatigue in the link between mobile phone addiction and mind wandering. The results were presented in Table 2. Mobile phone addiction showed a positive and significant total effect on mind wandering, with a regression coefficient of 0.630 (*p* < 0.001). Mobile phone addiction also showed a positive and significant effect on fatigue (*B* = 0.386, *p* < 0.001). When fatigue was included in the regression equation as a mediator, fatigue also showed a positive and significant effect on mind wandering (*B* = 0.502, *p* < 0.001), and mobile phone addiction still showed a positive and significant direct effect on mind wandering (*B* = 0.437, *p* < 0.001). Furthermore, the total effect, direct effect, and indirect effect were positively and significantly different from zero, as 95% bootstrapped confidence intervals for these effects did not include zero.

### 3.3. Testing for the Proposed Moderated Mediation Model

The SPSS macro PROCESS (model 8) was performed to estimate the moderating effect of rumination in the mediation model that has been supported by data. The results are shown in Table 3. After rumination was included as a moderator in the regression equation, mobile phone addiction still positively predicted fatigue (*B* = 0.368, *p* < 0.001) and mind wandering (*B* = 0.421, *p* < 0.001). Fatigue also showed a positive effect on mind wandering (*B* = 0.495, *p* < 0.001). These findings indicated that even if rumination was included as a moderator in the regression equation, fatigue could still mediate the relation between mobile phone addiction and mind wandering.

**Figure 2:**
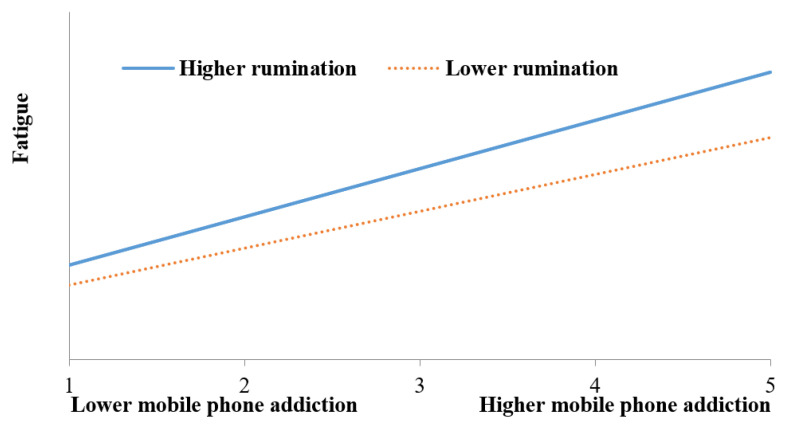
Rumination moderates the relationship between mobile phone addiction and fatigue.

**Figure 3:**
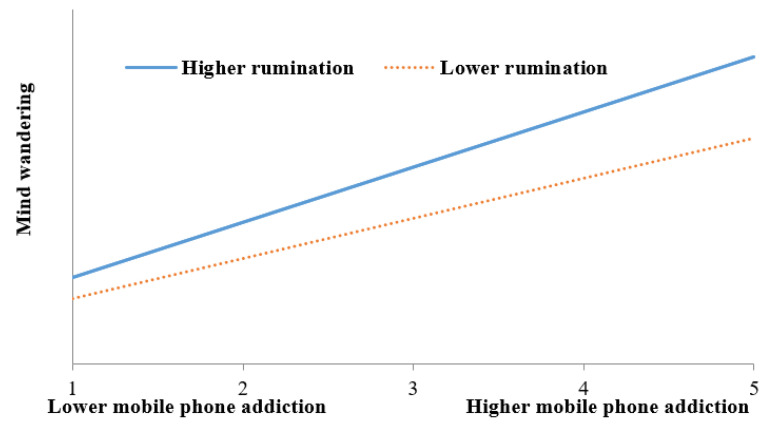
Rumination moderates the relationship between mobile phone addiction and mind wandering.

Besides, the interaction of mobile phone addiction and rumination showed not only a positive and significant effect on fatigue (*B* = 0.098, *p* < 0.05), but also a positive and significant effect on mind wandering (*B* = 0.142, *p* < 0.05). On this basis, two simple slope analyses were performed to decompose these two significant interaction effects. The results of simple slope analyses were plotted in Figure 2 and Figure 3. It can be seen from Figure 2 that whatever their level of rumination, the effect of mobile phone addiction on fatigue was positive and significant. The difference was that the effect of mobile phone addiction on fatigue was stronger for college students with high rumination (simple slope = 0.416, *t* = 14.737, *p* < 0.001) than for college students with low rumination (simple slope = 0.319, *t* = 10.095, *p* < 0.001). Figure 3 showed that the effect of mobile phone addiction on mind wandering was positive and significantly different from zero for both college students with low rumination and college students with high rumination. But this effect for college students with high rumination (simple slope = 0.698, *t* = 15.246, *p* < 0.001) was stronger than that for college students with low rumination Simple slope = 0.508, *t* = 10.171, *p* < 0.001).

Furthermore, the results of two conditional analyses showed that no matter what levels of rumination there are, all of the direct and indirect effects were positive and significantly different from zero. Specifically, for students with high rumination, both the direct effect of mobile phone addiction on mind wandering and the indirect effect of fatigue in this link were stronger.

## 4. Discussion

Considering that limited attention has been paid to the effect of mobile phone addiction on mind wandering and the mechanism underlying this link, this study, based on the previous research results and related theories, constructed a moderated mediation model exploring the relation between mobile phone addiction and mind wandering as well as answering the questions about how and for whom mobile phone addiction can affect individuals’ mind wandering. The moderated mediation analyses demonstrated that mobile phone addiction showed a positive association with mind wandering. Hypothesis 1 was supported. Fatigue mediated the association between mobile phone addiction and mind wandering. Hypothesis 2 was supported. Both the direct and indirect effects of mobile phone addiction on mind wandering would be exacerbated when individuals ruminate more about negative experiences drawn from excessive mobile phone use. Hypotheses 3a and 3b were supported. These findings not only revealed how (mediating mechanisms) and for whom (moderating mechanisms) mobile phone addiction resulted in mind wandering but also enlightened us that we could attenuate the potential adverse effects of mobile phone addiction on our attention quality by ruminating less about mobile phone related content and the negative experience caused by compulsive mobile phone use.

### 4.1. Mobile Phone Addiction and Mind Wandering

Consistent with a previous study [20,21,38], the current study indicated that mobile phone addiction will undermine individuals’ cognitive function. Specifically, mobile phone addiction could positively and significantly predict individuals’ mind wandering. This finding illustrated that, while mobile phones have brought many conveniences to our work and lives, we will pay a high price for excessive use, such as negative effects on psychological and behavioral adaptation [73]. Attention quality, as an important index of individual psychological adaptation, could not be spared from the adverse effects of mobile phone addiction [37]. Our finding validates and expands previous related research [20,21], which found that individuals with mobile phone addiction have similar cognitive problems to those with Internet game addiction and compulsive Internet use. Specifically, this is reflected in the two aspects of concentration and inhibition of mind wandering. From the perspective of focusing attention, mobile phone addiction predicted mind wandering, which was consistent with the executive-control failure hypothesis [22,23]. Mobile phone use will reduce an individual’s ability to control their attention and may even lead to an attention disorder [74]. Individuals suffering from mobile phone addiction have difficulty diverting their attention completely from mobile phones or mobile phone-related activities (e.g., fear of missing information, craving for games), and thus their minds wander more about activities on mobile phones in daily life [20,21]. From the perspective of curbing mind wandering, the rebound effect can be used to explain the daily mind wandering of mobile phone addicts. The rebound effect means that individuals who curb the idea of white bears will instead think more of them [75]. In daily life, mobile phone addicts may curb phone-related thoughts in order to better accomplish tasks or events. Due to the salience (e.g., the thinking and behavior of mobile phone addicts being dominated by their mobile phone activities) and withdrawal symptoms (e.g., mobile phone addicts will be caught up in the wave of unpleasant feelings, such as anxiety, when the activities on mobile phone are interrupted or blocked), they will instead think of mobile phones and produce mind wandering in their daily lives [76]. Moreover, mobile phone addicts are also distracted by thoughts or tasks other than their phones. According to the resource-control theory of mind-wandering [24], mobile phone addicts consume control resources by inhibiting mobile phone use. The decrease in control resources for mobile phone addicts represents insufficient control resources to inhibit other thoughts unrelated to other thoughts or tasks as well (e.g., negative emotions, social anxiety) [21,62] and produce mind wandering.

### 4.2. Fatigue as a Mediator

Fatigue has been considered an important factor leading to mind wandering [54,77]. Our findings, consistent with and expanding previous studies, showed that fatigue could act as a mediator linking mobile phone addiction to mind wandering. This finding indicated that fatigue induced by excessive or compulsive mobile phone use makes it difficult for individuals suffering from mobile phone addiction to concentrate on their target task and avoid mind wandering about mobile phone-related activities. Previous studies considered that mobile phones have the characteristics of integration, accessibility, and convenience, which makes the influence of mobile phone addiction on individuals’ psychological adaptation more profound and extensive [6,15]. Mobile phone addiction can be combined with several behavioral addiction subtypes, including social addiction, game addiction, short videos addiction, and information addiction [15,70,78]. This makes mobile phone addicts overwhelmed by plenty of activities that consume their energy and lead to fatigue. Previous studies showed that excessive mobile phone use based on social interaction (addiction to social networking sites) will not only make individuals experience higher cognitive load but also make individuals feel discomfort, be extremely tired, and exhibit other symptoms of fatigue [10,43,48]. It happens that there is a similar case. Mobile phone-based game addiction and short video addiction will consume individuals’ rest time, especially the time in bed, which will not only put individuals in a state of exhaustion but also lead to physical and psychological fatigue.

There is also a natural link between fatigue and mind wandering, which also provides empirical evidence for the resource-control theory of mind-wandering [24]. Good attention quality needs a good physical and psychological state to maintain. Energetic individuals are more likely to avoid the interference of external stimuli and inner awareness activities and focus on the target task. Physically and mentally exhausted individuals will fail to shield the interference of external stimuli and control their conscious activities effectively [79]. Previous studies showed that fatigue will consume individuals’ self-control resources, resulting in low levels of self-control ability or executive control ability [79]. According to the executive-control failure hypothesis and the resource-control theory of mind-wandering [22,23,24,25], mind wandering represents a failure of self-control or executive control. Empirical studies also showed a significant positive correlation between fatigue and mind wandering [77]. In conclusion, the fatigue induced by excessive use of mobile phones could mediate the link between mobile phone addiction and mind wandering.

### 4.3. Rumination as a Moderator

In addition, both the direct predictive effect of mobile phone addiction on mind wandering and the indirect effect of fatigue varied with individuals’ levels of rumination. Specifically, both the direct effect that mobile phone addiction itself exerted on mind wandering and the mediating effect of fatigue were all stronger for individuals with a higher level of rumination. These results indicated that rumination, as one of the negative psychological traits or non-adaptive response styles, could amplify or aggravate the potential adverse effects of mobile phone addiction on our subjective experience (fatigue) and attention quality (mind wandering). Given that mobile phone addicts with higher levels of rumination suffered more harm from excessive mobile phone use, more attention should be paid to guiding them to consciously reshape their response style so as to reduce the severity of fatigue and mental wandering caused by mobile phone use.

This finding was consistent with previous research that found that rumination moderated the effect of mobile phone addiction on individuals’ emotional adaptation (affect balance, fatigue, and depression) and sleep quality [15,53]. According to response style theory [80,81], compared with mobile phone addicts with low levels of rumination, those with high levels of rumination may be involved in more emotional problems and other psychological adaptation problems, such as emotional imbalance, fatigue, depression, and poor sleep quality, due to their high tendency to ruminate about their experience and negative emotions drawn from excessive or compulsive mobile phone use. Rumination, consisting of emotional rumination and cognitive rumination, could aggravate the adverse effect of mobile phone addiction on fatigue and mind wandering through both the emotional process and the cognitive process. Previous studies have shown that mobile phone addiction is positively associated with depression, anxiety, stress, and other negative emotions [11,39]. Emotional rumination (the tendency of individuals to repeatedly experience negative emotions) [82] may moderate the emotional regulation strategies of mobile phone addicts to deal with the negative emotions caused by excessive mobile phone use. Instead of taking effective emotional regulation strategies to deal with negative emotions or the state in which they feel discomfort and are extremely tired, mobile phone addicts with high levels of rumination may be invaded by these negative experiences and get involved in more negative emotions. Given that negative emotion has a positive relationship with both fatigue and mind wandering [27,33,40,83,84], mobile phone addicts who have high levels of rumination may experience more and get distracted by these negative experiences. Besides, cognitive rumination may serve as a negative thinking style and moderate the cognitive process that mobile phone addicts use to deal with the negative experiences drawn from excessive mobile phone use. Since the negative associative memory networks of individuals with high rumination are more likely to be activated by negative experiences, it is difficult for them to concentrate and take effective measures to deal with the negative experiences they faced [85]. Mobile phone addicts with high levels of rumination may fail to find effective problem-solving strategies and take action to deal with the negative experience caused by excessive mobile phone use. This will not only trap them in these negative experiences for an extended period of time, resulting in fatigue, but it will also seize their attention to deal with these negative experiences. Moreover, rumination, characterized by uncontrollable thinking about the causes and adverse consequences of what has happened, will not only distract individuals’ attention and induce mental wandering but also consume a lot of energy and make individuals feel tired. Therefore, mobile phone addicts with high levels of rumination will experience more fatigue and have a higher level of distraction or mind wandering.

### 4.4. Limitations and Implications

Although this study filled the gap between mobile phone addiction and individuals’ attention quality by revealing how and for whom mobile phone addiction leads to mind wandering, this study still has limitations. First, unlike previous cross-sectional studies, the present cross-sectional study was unable to draw a rigorous and anticipated causal relationship. Longitudinal or experimental studies should be conducted to examine or explore the causal direction among mobile phone addiction, fatigue, and mind wandering. Ingenious interventional designs should also be employed in future research to test the moderating effect of rumination by comparing the changes in the effects of mobile phone addiction on fatigue and mind wandering before and after intervening. Second, although the investigators emphasized the anonymity of the survey and the confidentiality of the survey results before the participants began to fill the self-report questionnaires, the scientificity, precision, and effectiveness of the data in this study may still be compromised or restricted by social desirability bias. To handle this potential problem, future studies should adopt multi-rater assessment to collect information about participants not just from their self-report but also from other important sources, such as their parents, teachers, and peers. Third, although 1811 college students from three regular universities in three cities located in southwest (Xihua and Jingzhou) or southeast (Hangzhou) China, the generalizability of the study’s findings will still be limited by the representativeness of the sample. The conclusions of this study should be interpreted with caution when generalizing to adolescents, wage-earners, and other populations from different cultures. Furthermore, because all college students are nested in classes, majors, colleges, and universities, the atmosphere of class, major, college, and university may also moderate the relation among mobile phone addiction, fatigue, and mind wandering as well as the moderating effect of rumination. However, limited by research resources, this study failed to recruit more participants from more schools, colleges, majors, and classes and build hierarchical linear modeling to examine the effect of the atmosphere of class, major, college, and university.

Despite these shortcomings, this study has some valuable contributions. First, the present study filled the gap left by previous studies by drawing attention to the effect of mobile phone addiction on individuals’ mind wandering. Meanwhile, the study extends the influence of compulsive Internet use, Internet game addiction, and other problematic Internet use on mind wandering to the field of mobile phone addiction [20,21]. Second, this study provided empirical support for the executive control failure hypothesis [22,23] and the resource-control theory of mind-wandering [24]. Specifically, fatigue, a typical manifestation of a lack of control over resources, is significantly associated with individual wandering. Moreover, the present study not only responded to the question about how or why mobile phone addiction leads to mind wandering by revealing the mediating effect of fatigue but also answered the question about for whom the effects of mobile phone addiction on fatigue and mind wandering were stronger by examining the moderating effects of rumination in these links.

In addition, practical implications could also be drawn from our findings. First, given that mobile phone addiction was positively associated with mind wandering, to reduce mind wandering and focus on our task, we should consciously and moderately use mobile phones to serve our work and lives rather than relying on them excessively. Second, given that fatigue bridges the link between mobile phone addiction and mind wandering, to reduce the possibility of mind wandering after we overuse mobile phones, we could break the bridge formed by fatigue by relieving our fatigue through mindfulness meditation or music-based relaxation training. Previous studies have demonstrated that mindfulness meditation or music-based relaxation training could help us release our fatigue [86,87,88]. Third, the moderating effect of rumination revealed that educators or parents should pay closer attention to individuals who have a high level of rumination and guide them consciously reduce their use of mobile phones responsibly to reduce the risk of fatigue and mind wandering from the source. Besides, previous studies have shown that mindfulness training and other intervention programs could weaken individuals’ levels of rumination and mind wandering [35,89]. Individuals with a high level of rumination could also alleviate their frequency of mind wandering and the adverse effect of mobile phone addiction on them by participating in mindfulness training and alleviating their level of rumination.

## 5. Conclusions

This study attempted to fill in the research gap on the effects of mobile phone addiction on mind wandering. The results found that mobile phone addiction was not only significantly positively associated with wandering but was also related to the mediating effect of fatigue. Meanwhile, the direct and indirect effects of mobile phone addiction on mind wandering could be moderated by rumination. Specifically, both the direct and indirect effects were stronger for students with high rumination. Therefore, we need to pay more attention to the adverse effects of mobile phone addiction on individuals’ states of consciousness. The potential adverse effects can be mitigated by reducing rumination about mobile phone-related content and the negative experiences of compulsive mobile phone use. Future research should focus on the relationship between mobile phone addiction and individual attention and examine ways to mitigate this effect (e.g., mindfulness).

## Figures and Tables

**Table 1:** Descriptive statistics and interrelations among some of the observed variables.

Variables	*M*	*SD*	1	2	3	4	5	6
1. Age	19.740	1.295	1					
2. Years of mobile phone usage	5.280	2.356	0.196 **	1				
3. Mobile phone addiction	2.603	0.609	−0.084 **	0.070 **	1			
4. Fatigue	2.428	0.592	−0.021	0.032	0.391 **	1		
5. Mind wandering	3.692	0.903	−0.023	0.020	0.416 **	0.442 **	1	
6. Rumination	2.318	0.499	−0.029	0.005	0.163 **	0.117 **	0.106 **	1

** *p* < 0.01.

**Table 2:** Regression results for the mediating effect of fatigue (mediation model).

Model									
Model 1: Total effect model									
*R*	*R* ^2^	*F*	*df*_1_	*df*_2_	*p*	*B*	*SE*	*t*	*p*
0.42	0.18	70.81	5	1805	<0.001				
Constant						2.211 ***	0.418	5.285	<0.001
Gender						−0.091 *	0.023	−2.186	<0.05
Age						−0.002	0.023	−0.078	>0.05
Grade						0.017	0.035	0.486	>0.05
Years of mobile phone usage						−0.002	0.009	−0.244	>0.05
Mobile phone addiction						0.630 ***	0.034	18.690	<0.001
Model 2: Mediator variable model									
*R*	*R* ^2^	*F*	*df*_1_	*df*_2_	*p*	*B*	*SE*	*t*	*p*
0.40	0.16	62.22	5	1805	<0.001				
Constant						1.841 ***	0.267	6.904	<0.001
Gender						−0.039	0.028	−1.399	>0.05
Age						−0.025	0.015	−1.670	>0.05
Grade						0.058 **	0.022	2.646	<0.01
Years of mobile phone usage						0.002	0.006	0.327	>0.05
Mobile phone addiction						0.386 ***	0.022	17.529	<0.001
Model 3: Dependent variable model									
*R*	*R* ^2^	*F*	*df*_1_	*df*_2_	*p*	*B*	*SE*	*t*	*p*
0.51	0.27	101.18	6	1084	<0.001				
Constant						1.288 **	0.413	3.120	<0.01
Gender						−0.071	0.039	−1.816	>0.05
Age						0.011	0.022	0.475	>0.05
Grade						−0.012	0.033	−0.377	>0.05
Years of mobile phone usage						−0.003	0.008	−0.370	>0.05
Mobile phone addiction						0.437 ***	0.036	12.237	<0.001
Fatigue						0.502 ***	0.038	13.311	<0.001
						*B*	Boot SE	BootLLCI	BootULCI
Total effect of mobile phone addiction on mind wandering						0.630	0.034	0.564	0.696
Direct effect of mobile phone addiction on mind wandering						0.437	0.036	0.367	0.507
Indirect effect of fatigue						0.194	0.018	0.159	0.232

* *p* < 0.05. ** *p* < 0.01. *** *p* < 0.001. Unstandardized regression coefficients are reported. Bootstrap sample size = 5000. LL = low limit, CI = confidence interval, UL = upper limit.

**Table 3:** Regression results for the conditional indirect effects (moderated mediation).

Model									
Model 1: Mediator variable model									
*R*	*R* ^2^	*F*	*df_1_*	*df_2_*	*p*	*B*	*SE*	*t*	*p*
0.40	0.16	0.294	7	1803	<0.001				
Constant						2.862 ***	0.260	11.001	<0.001
Gender						−0.041	0.028	−1.475	>0.05
Age						−0.026	0.015	−1.774	>0.05
Grade						0.062 **	0.022	2.832	<0.01
Years of mobile phone usage						0.022	0.006	0.387	>0.05
Mobile phone addiction						0.368 ***	0.022	16.570	<0.001
Rumination						0.073 **	0.027	2.672	<0.01
Mobile phone addiction × Rumination						0.098 *	0.041	2.408	<0.05
Model 2: Dependent variable model									
*R*	*R* ^2^	*F*	*df_1_*	*df_2_*	*p*	*B*	*SE*	*t*	*p*
0.52	0.27	75.20	8	1802	<0.001				
Constant						2.443	0.408	5.982	<0.001
Gender						−0.072	0.039	−1.823	>0.05
Age						0.010	0.022	0.442	>0.05
Grade						−0.010	0.033	−0.305	>0.05
Years of mobile phone usage						−0.003	0.008	−0.305	>0.05
Fatigue						0.495 ***	0.038	13.073	<0.001
Mobile phone addiction						0.421 ***	0.036	11.692	<0.001
Rumination						0.042	0.038	1.111	>0.05
Mobile phone addiction × Rumination						0.142 *	0.061	2.322	<0.05
Conditional direct effect analysis at values of rumination (*M* ± *SD*)									
						*B*	SE	LLCI	ULCI
*M −* 1*SD* (1.819)						0.350	0.049	0.255	0.446
*M* (2.318)						0.421	0.036	0.351	0.492
*M +* 1*SD* (2.817)						0.492	0.046	0.402	0.582
Conditional indirect effect analysis at values of rumination (*M* ± *SD*)									
						*B*	Boot SE	BootLLCI	BootULCI
*M –* 1*SD* (1.819)						0.158	0.020	0.122	0.200
*M* (2.318)						0.182	0.018	0.150	0.219
*M +* 1*SD* (2.817)						0.206	0.021	0.168	0.250

* *p* < 0.05. ** *p* < 0.01. *** *p* < 0.001. Unstandardized regression coefficients are reported. Bootstrap sample size = 5000. LL = low limit, CI = confidence interval, UL = upper limit.

## Data Availability

The data of this study are available from the corresponding author upon reasonable request.
